# Comparison of clinical outcomes between nurse practitioner and registrar-led medical emergency teams: a propensity-matched analysis

**DOI:** 10.1186/s13054-021-03534-4

**Published:** 2021-03-22

**Authors:** Sachin Gupta, Mayurathan Balachandran, Gaby Bolton, Naomi Pratt, Jo Molloy, Eldho Paul, Ravindranath Tiruvoipati

**Affiliations:** 1 Department of Intensive Care Medicine, Peninsula Health, Melbourne, Australia; 2 Faculty of Medicine, Nursing and Health Sciences, Monash University, Melbourne, Australia; 3 ANZIC-RC, Department of Epidemiology and Preventive Medicine, School of Public Health and Preventive Medicine, Monash University, 553 St Kilda Road, Melbourne, VIC 3004 Australia; 4 Department of Intensive Care Medicine, Frankston Hospital, Frankston, VIC 3199 Australia

**Keywords:** Medical emergency team, Deterioration, Mortality, Discharge

## Abstract

**Objective:**

Medical emergency teams (MET) are mostly led by physicians. Some hospitals are currently using nurse practitioners (NP) to lead MET calls. These are no studies comparing clinical outcomes between these two care models. To determine whether NP-led MET calls are associated with lower risk of acute patient deterioration, when compared to intensive care (ICU) registrar (ICUR)-led MET calls.

**Methods:**

The composite primary outcome included recurrence of MET call, occurrence of code blue or ICU admission within 24 h. Secondary outcomes were mortality within 24 h of MET call, length of hospital stay, hospital mortality and proportion of patients discharged home. Propensity score matching was used to reduce selection bias from confounding factors between the ICUR and NP group.

**Results:**

A total of 1343 MET calls were included (1070 NP, 273 ICUR led). On Univariable analysis, the incidence of the primary outcome was higher in ICUR-led MET calls (26.7% vs. 20.6%, *p* = 0.03). Of the secondary outcome measures, mortality within 24 h (3.4% vs. 7.7%, *p* = 0.002) and hospital mortality (12.7% vs. 20.5%, *p* = 0.001) were higher in ICUR-led MET calls. Propensity score-matched analysis of 263 pairs revealed the composite primary outcome was comparable between both groups, but NP-led group was associated with reduced risk of hospital mortality (OR 0.57, 95% CI 0.35–0.91, *p* = 0.02) and higher likelihood of discharge home (OR 1.55, 95% CI 1.09–2.2, *p* = 0.015).

**Conclusion:**

Acute patient deterioration was comparable between ICUR- and NP-led MET calls. NP-led MET calls were associated with lower hospital mortality and higher likelihood of discharge home.

**Supplementary Information:**

The online version contains supplementary material available at 10.1186/s13054-021-03534-4.

## Introduction

Over the last two decades, establishment of rapid response systems (RRS) in healthcare services has led to a reduction of in-hospital mortality and incidence of in-hospital cardiac arrests [1]. Reductions in the incidence of cardiac arrests have been linked with an increase in “dose” of medical emergency team (MET) calls [2]. The composition and the title of the team responding to MET calls varies between different jurisdictions. These teams are called as critical care outreach teams, MET teams, patient at risk teams and high capability teams in different countries [3]. The teams are largely led by physicians [4, 5]. The nurse-led MET teams are second most common model reported in the literature [6].

Physicians attending these MET calls generally have other defined roles. Attendance at MET calls was known to cause significant disruption to the critical care physicians’ usual activities [7, 8]. To reduce disruptions to the critical care staff with defined roles, a small proportion of healthcare services have introduced trained nurse practitioners (NP) specifically employed to lead MET call response. The growing expertise in acute management of deteriorating patients outside the ICUs and the evolving role of NPs has resulted in examination of the role of NPs in leading MET response [9–14]. To our knowledge, clinical outcomes between NP-led MET calls and intensive care unit registrar, a junior doctor in training (ICUR)-led MET calls have not been compared. The aim was to compare clinical outcomes including subsequent acute deterioration as evidenced by recurrence of MET call, occurrence of code blue or ICU admission within 24 h of first MET call between NP- and ICUR-led MET calls (NPMET study). We hypothesized that the primary composite outcome comprising recurrence of MET call, occurrence of code blue or admission to ICU within 24 h of the MET calls would not be different between NP- and ICUR-led MET calls.

## Methods

### Ethics approval

The NPMET study was identified as a quality assurance activity and was approved by study site Research Ethics Committee (approval no. QA17PH44).

### Study design and setting

Retrospective study of all MET calls between June 1, 2016, and March 9, 2018, in an Australian tertiary ICU.

### Patients

Admitted patients who had acute clinical deterioration to warrant a MET call during the study period.

### Sources of data

All rapid response calls (RRC) are entered in Riskman database. All patient medical records are scanned in a digital medical record system. Both these databases will be interrogated to find MET calls, eligible for inclusion. Data regarding physiological observations are extracted from RRC observation form, scanned into digital medical record system. Demographic data, diagnoses, discharge destination and hospital outcome will be derived from iPM. Health information services (HIS) at Peninsula health, scan all inpatient medical records to determine comorbidities and Charleston Comorbidity Index (CCI). This information will be extracted from HIS database. Acute Physiological and Chronic Health Evaluation score II and III recorded in COMET database, for all patients admitted to ICU. These data along with admission and discharge time, for patients admitted to ICU, will be extracted using COMET database. Data for treatment provided, outcome and alteration in goals of care are entered into Riskman database and were extracted for the study.

### Rapid response system (RRS)

RRS at the study site comprises two-tiered efferent limb, (1) comprising single criterion-triggered MET response for patients meeting physiological observation thresholds or staff concern triggers (Additional file 1: E-Table 1), and (2) code blue response for patients who were unresponsive, had respiratory or cardiac arrest, threatened airway or anaphylaxis.

The two clinical leads from the Critical Care Liaison nursing team participate in all MET calls and code blue responses when on duty. They each have greater than 20 years of critical care nursing experience and have completed postgraduate study in Critical Care Nursing and Master of Nursing (Nurse Practitioner). These two members have received endorsement from the Nursing and Midwifery Board of Australia to practice as Nurse Practitioners. The nurse practitioners have an advanced scope of practice which enables them to prescribe medication within a hospital approved formulary, order and interpret diagnostic investigations, refer to specialist teams and commence treatment. Their scope of practice includes advanced life support interventions including insertion of intra-osseous access, laryngeal mask airways and code team leadership. NP service was provided by two nurse practitioners and was available to be rostered for 8 h between 08:00 and 22:00 h (08:00–16:00 or 14:00–22:00), all days of the week including weekends. When NPs were not rostered, ICUR were assigned the role of attending and leading MET calls. All ICURs had at least four years of work experience after completing under-graduation, at least 9 months of which was spent either in ICU or emergency department resuscitation area under supervision of specialist consultants. All ICURs were either advanced emergency department trainees or registered as a trainee with College of Intensive Care Medicine (CICM). All ICURs received formal training toward recognizing and responding to critical patient deterioration, vascular access procedures and basic airway management techniques. Majority of ICURs were trained to perform advanced airway management including endotracheal intubation. Both NPs and ICUR could receive guidance from an ICU consultant physically present during business hours (08:00 to 18:00 h) and were on call out of hours (18:00 to 08:00 h).

### Inclusion criteria for MET calls

MET calls were included in the analysis if.

- They were the first (index) MET call for that hospital admission; and
- occurred between June 1, 2016, and March 9, 2018; and
- were led by either ICUR or NP.

### Exclusion criteria for MET calls

MET calls were excluded from the analysis if both NP and ICUR attended, neither NP nor ICUR attended, no record of attendees in MET call record or patients not admitted to hospital.

### Outcomes

#### Primary outcome

Primary outcome is a composite outcome comprising of either recurrence of MET call, occurrence of code blue or ICU admission within 24 h of index MET call.

#### Secondary outcomes

Secondary outcomes were mortality within 24 h of index MET call, length of hospital stay, hospital mortality and proportion of patients discharged home.

### Statistical analyses

Continuous variables were summarized using mean and standard deviation (SD) or median and interquartile range (IQR) depending on the underlying distribution of the data. Categorical variables were reported as counts and percentages. Comparisons between groups (NP vs. ICUR) were made using the Student’s t test for normally distributed continuous variables, Wilcoxon rank-sum test for non-normally distributed continuous variables and Chi-square or Fisher’s exact test as appropriate for categorical variables.

Propensity score matching was used to reduce selection bias from confounding factors between the ICUR and NP group. The individual propensities for being in the ICUR-led group were estimated with the use of a multivariable logistic regression model that included age, Charlson Comorbidity Index (CCI), weekend (Saturday and Sunday) occurrence of MET call, out of hours occurrence of MET call, “not for resuscitation” plan prior to MET call, triggering of MET call due to oxygen saturation < 90% and diagnoses (medical vs. surgical) as the predictor variables. This propensity score was used to match patients managed by ICUR to those managed by NP using a one-to-one nearest neighboring matching with a caliper width of 0.15 times the standard deviation. Standardized differences were calculated to assess the balance of covariates between ICUR and NP groups. Primary and secondary outcomes were compared between ICUR- and NP-led groups using conditional logistic regression taking into account the matched design with results reported as odds ratios (OR) and 95% confidence intervals (95% CI). Sensitivity analysis was also performed using a multivariable logistic regression model that included the same covariates as the propensity score model to ensure robustness of the primary analysis.

All reported p values are two-sided, and a p value less than 0.05 was considered to indicate statistical significance.

## Results

**Fig. 1:**
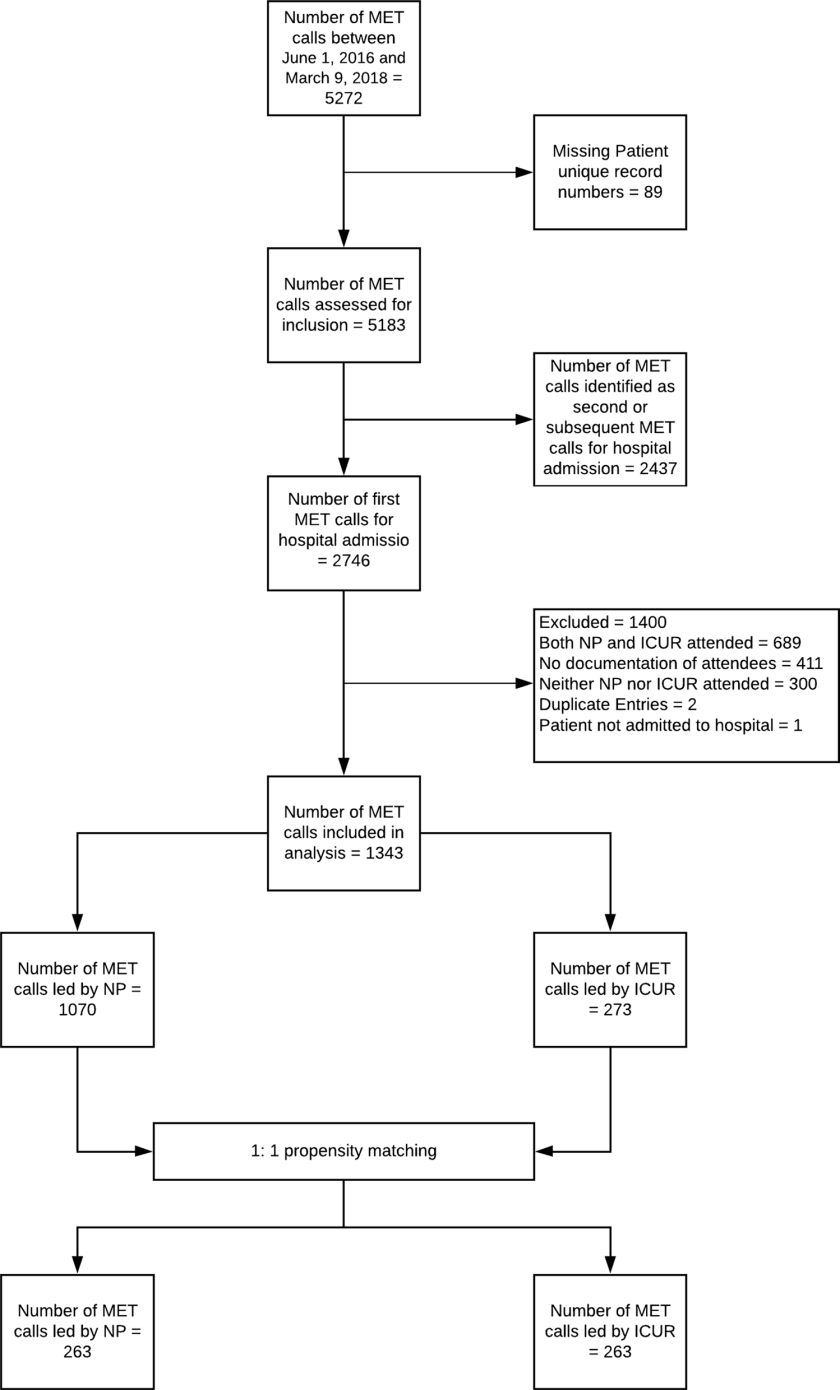
Flow diagram of the MET calls included in analysis

In total, 5272 MET calls occurred between June 1, 2016, and March 9, 2018. Of these, 1343 (25.5%) MET calls were included in the analysis (Fig. 1). The main reasons for exclusions included MET calls identified as second or subsequent (*n* = 2437) and attendance of MET calls by both NPs and ICUR (*n* = 689). The other reasons for exclusion are provided in Fig. 1.

Baseline characteristics of both NP-led and ICUR-led MET calls are presented (Table 1). As shown in Table 1, age, gender, and CCI scores were comparable between both groups. Cardiovascular diagnoses and medical patients were significantly more common in ICUR-led MET calls, while surgical patients were higher in NP-led MET calls (Table 1).

**Table 1:** Comparison of baseline characteristics between NP- and ICUR-led MET calls

	All patients (*n* = 1343)	NPMET cohort				Propensity-matched cohort		
		NP led (*n* = 1070)	ICUR led (*n* = 273)	*p* value	Std. Diff.^a^	NP led (*n* = 263)	ICUR led (*n* = 263)	Std. Diff.^a^
Age (SD)	69 (18.5)	69.3 (18.3)	67.7 (19.1)	0.2	8.6	68.2 (18.5)	67.4 (19.2)	4.2
Female (%)	717 (53.4)	564 (52.7)	153 (56)	0.3	6.6	136 (51.7)	147 (55.9)	8.4
*Admission diagnosis system*								
Respiratory system (%)	213 (15.9)	174 (16.3)	39 (14.3)	0.4	5.6	51 (19.4)	36 (13.7)	15.4
Cardiovascular system (%)	185 (13.8)	131 (12.2)	54 (19.8)	0.001	20.8	34 (12.9)	51 (19.4)	17.7
Nervous system (%)	196 (14.6)	154 (14.4)	42 (15.4)	0.7	2.8	38 (14.4)	41 (15.6)	3.4
Gastrointestinal system (%)	214 (15.9)	176 (16.4)	38 (13.9)	0.3	7.0	44 (16.7)	36 (13.7)	8.3
Genitourinary system (%)	111 (8.3)	91 (8.5)	20 (7.3)	0.5	4.4	21 (8.0)	20 (7.6)	1.5
Other (%)	424 (31.6)	344 (32.1)	80 (29.3)	0.4	6.1	75 (28.5)	79 (30)	3.3
*Diagnostic category*								
Medical (%)	870 (65)	677 (63.4)	193 (71.2)	0.016	16.7	176 (66.9)	186 (70.7)	8.2
Surgical (%)	438 (32.7)	363 (34)	75 (27.7)	0.048	13.7	84 (31.9)	74 (28.1)	8.3
Obstetrics and gynecology (%)	31 (2.3)	28 (2.6)	3 (1.1)	0.1	11.1	3 (1.1)	3 (1.1)	0
Charlson Comorbidity Index (IQR)	1 (0–3)	1 (0–3)	1 (0–3)	0.2	4.6	1 (0–3)	1 (0–3)	12.2

^a^Std. Diff (standardized difference) is the mean difference divided by the pooled SD, expressed as a percentage

Out of 1343 patients, 1322 (98.4%) had triggers recorded in MET call documentation (Table 2). Out of hours MET calls were higher in ICUR-led MET calls, whereas weekend MET calls were distributed similarly in both groups (Table 2). The triggers for MET calls were comparable between both groups but for oxygen saturation < 90%, which was more common in ICUR-led MET calls (15% vs. 9.9%, *p* = 0.02).

**Table 2:** Comparison of characteristics of MET calls and patients admitted to ICU

	NPMET cohort					Propensity-matched cohort		
	All (*n* = 1343)	NP led (*n* = 1070)	ICUR led (*n* = 273)	*p* value	Std. diff.^e^	NP led (*n* = 263)	ICUR led (*n* = 263)	Std. Diff.^e^
*Timing*								
Out of hours (6 PM to 8 AM)	477 (35.5)	237 (22.1)	240 (87.9)	< 0.0001	176.3	231 (87.8)	231 (87.8)	0
Weekend (Saturday/Sunday)	342 (25.5)	263 (24.6)	79 (28.9)	0.14	9.7	71 (27.0)	75 (28.5)	3.4
*Trigger*^*@*^								
Altered conscious state	169/1322 (12.8)	144/1056 (13.6)	25/266 (9.4)	0.06	13.2	28 (10.6)	25 (9.5)	3.6
Arrhythmia	5/1322 (0.4)	3/1056 (0.3)	2/266 (0.8)	0.3	6.5	1 (0.4)	2 (0.8)	5
Heart rate < 40/min	8/1322 (0.6)	6/1056 (0.6)	2/266 (0.8)	0.7	2.3	1 (0.4)	2 (0.8)	5
Heart rate > 130/min	222/1322 (17)	176/1056 (16.7)	46/266 (17.3)	0.8	1.6	57 (21.7)	45 (17.1)	11.7
Obstructed/threatened airway	8/1322 (0.6)	5/1056 (0.5)	3/266 (1.1)	0.2	7.1	1 (0.4)	3 (1.1)	8.4
Staff concern	141/1322 (10.7)	115/1056 (10.9)	26/266 (9.8)	0.6	3.6	30 (11.4)	25 (9.5)	6.2
Respiratory distress	88/1322 (6.7)	65/1056 (6.2)	23/266 (8.6)	0.2	9.2	18 (6.8)	22 (8.4)	6
Respiratory rate < 8/min	4/1322 (0.3)	4/1056 (0.4)	0/266	0.6	8.7	0 (0)	0 (0)	0
Respiratory rate > 30 or 36/min	52/1322 (3.9)	45/1056 (4.3)	7/266 (2.6)	0.2	9.3	8 (3.0)	7 (2.7)	1.8
Oxygen saturation < 90%	145/1322 (11)	105/1056 (9.9)	40/266 (15)	0.02	15.5	25 (9.5)	40 (15.2)	17.3
SBP^d^ < 90 mm Hg	267/1322 (20.2)	213/1056 (20.2)	54/266 (20.3)	1	0.2	44 (16.7)	54 (20.5)	9.8
SBP^d^ > 180- or 200 mm Hg	169/1322 (12.8)	143/1056 (13.5)	26/266 (9.8)	0.1	11.6	40 (15.2)	26 (9.9)	16
Seizures	37/1322 (2.8)	27/1056 (2.6)	10/266 (3.8)	0.29	6.8	9 (3.4)	10 (3.8)	2.1
Urine output less than 50 ml/4 h	7/1322 (0.5)	5/1056 (0.5)	2/266 (0.8)	0.6	3.6	1 (0.4)	2 (0.8)	5
*Observations at onset of MET call*^a^								
Heart rate (*n* = 1067)	100 (31.8)	99 (30.7)	105 (36)	0.02	18.5	106 (32.2)	105 (35.9)	2.9
Respiratory rate (*n* = 738)	23 (8.7)	23 (8.8)	24 (8.3)	0.4	8.2	22.8 (8.8)	23.7 (8.3)	10.5
SBP (*n* = 1059)	132 (42.3)	132 (42)	130 (44.1)	0.5	4.6	137 (43)	130 (44.5)	16
Oxygen saturation^b^ (*n* = 980)	92 (8.4)	93 (8.1)	91 (9.5)	0.002	23.9	92.3 (7.4)	90.7 (9.6)	18.7
GCS^c^ (*n* = 46)	14 (9–15)	14 (9–15)	14 (7–15)	0.8	17.4	12.3 (4.5)	12.4 (3.7)	2.4
Hospital LOS prior to MET call^d^	1.8 (0.6–4.1)	1.8 (0.7–4.1)	1.8 (0.6–4.4)	0.8	3.0	3.1 (4.8)	3.8 (5.6)	13.5
Hospital LOS prior to MET call < 24 h	483 (36)	387 (36.2)	96 (35.2)	0.8	2.1	(57.4)	(64.3)	14.2
NFR documented prior to MET	412 (30.7)	321 (30.8)	91 (33.5)	0.3	7.3	(27.8)	(33.1)	11.5
*Patients admitted to ICU within 24 h of MET call*								
APACHE II score	16.8 (6.2)	17.1 (6.2)	16.1 (6.5)	0.4	15.8	19.2 (7.6)	16.4 (6.5)	39.5
APACHE III score	61.4 (22.3)	61.5 (23)	61.3 (21)	1	0.1	64.3 (27.6)	62.6 (20.7)	7
ICU length of stay	2.2 (1.2–3.5)	2 (1.1–3.6)	2.4 (1.3–3.4)	0.9	6.1	2.8 (3.5)	4.1 (6.9)	
ICU mortality	10 (8.1)	7 (8.5)	3 (7.3)	0.8	4.4	2 (13.3)	3 (7.7)	

*SBP *systolic blood pressure, *LOS* length of stay

^a^First observations recorded in MET call observation sheet with number of MET calls with each observation recorded in parentheses, expressed as mean values with SD in parenthesis

^b^Percentage of hemoglobin saturated with oxygen as recorded by pulse oximetry

^c^Expressed as median with IQR in parentheses

^d^Described as median hospital length of stay prior to triggering MET call, with IQR expressed in parentheses

APACHE—Acute Physiology and Chronic Health Evaluation

^e^Standardized difference (Std. diff.) is the mean difference divided by the pooled SD, expressed as a percentage

Of the observations compared at the onset of MET calls, heart rate (105 vs. 99, *p* = 0.02) and oxygen saturation level (91% vs. 93%, *p* = 0.002) were different between the groups (Table 2).

There was no statistically significant difference in hospital length of stay prior to MET call, MET calls occurring within 24 h of admission and not for resuscitation status between both groups (Table 2). The severity of illness, length of ICU stay and ICU mortality of patients admitted to ICU were comparable between both groups (Table 2).

A comparison of interventions performed during attendance for MET call is presented in Table 3. Intravenous cannulation (24.1% vs. 15.4%, *p* = 0.002), fluid bolus administration (33.4% vs. 26.5%, *p* = 0.03) and performing ECG (58.6% vs. 42.6%, *p* < 0.001) were more frequent during NP-led MET calls. Occurrence of change in resuscitation status was similar in both groups (16.2% in NP led vs. 13.6% in ICUR led, *p* = 0.3).

**Table 3:** Interventions recorded during MET calls

	All (*n* = 1343)	NP led (*n* = 1070)	ICUR led (*n* = 273)	*p* value
IV cannulation	300 (22.4)	258 (24.1)	42 (15.4)	0.002
Fluid bolus	429 (32)	357 (33.4)	72 (26.5)	0.03
ECG	742 (55.4)	627 (58.6)	115 (42.6)	< 0.001
Arterial blood gas analysis	280 (20.9)	222 (20.7)	58 (21.6)	0.8
Body fluid or blood cultures	153 (11.4)	131 (12.2)	58 (21.6)	0.77
Bag and mask ventilation	2 (0.2)	1 (0.1)	1 (0.4)	1
Endotracheal intubation	1 (0.1)	0	1 (0.4)	0.2
Change in resuscitation plans after MET call	211 (15.7)	174 (16.2)	37 (13.6)	0.3

The primary and secondary outcomes in both groups are presented in Table 4. NP-led MET calls were associated with lesser occurrence of the composite outcome as compared to ICUR-led MET calls (20.6% vs. 26.7%; *p* = 0.03). There were fewer ICU admissions within 24 h of index MET call in NP-led MET calls as compared to ICUR-led MET calls (7.7% vs. 15%; *p* < 0.0001).

**Table 4:** Comparison of outcomes between NP-led versus ICUR-led MET calls

	All (*n* = 1343)	NP led (*n* = 1070)	ICUR led (*n* = 273)	Odds ratio (95% CI)	*p value*
*Primary outcome*					
Composite outcome	293 (21.8)	220 (20.6)	73 (26.7)	0.71 (0.52–0.96)	0.028
*Components of composite outcome*					
At least one MET call within 24 h	204 (15.2)	163 (15.2)	41 (15)	1.02 (0.70–1.47)	0.93
Code blue within 24 h	4 (0.3)	3 (0.3)	1 (0.4)	0.76 (0.08–7.38)	0.817
ICU admission within 24 h	123 (9.2)	82 (7.7)	41 (15)	0.47 (0.31–0.70)	< 0.001
Secondary outcomes					
Mortality within 24 h	57 (4.2)	36 (3.4)	21 (7.7)	0.42 (0.24–0.73)	0.002
Hospital mortality	192 (14.3)	136 (12.7)	56 (20.5)	0.56 (0.40–0.80)	0.001
Hospital length of stay	7.4 (4.1–14.2)	7.54 (4.1–14.2)	7.2 (4–14.1)	1 (0.99–1.01)	0.766
Discharged home	662 (49.3)	538 (50.3)	124 (45.4)	1.22 (0.93–1.59)	0.15

NP-led MET calls were also associated with lower mortality within 24 h of index MET call (3.4% vs. 7.7%, *p* = 0.002) and lower hospital mortality (12.7% vs. 20.5%, *p* = 0.001). The hospital length of stay (median 7.5 days vs. 7.2 days, *p* = 0.9) and proportion of patients discharged home (50.3% vs. 45.4%, *p* = 0.15) were comparable.

### Propensity-matched analysis

The propensity-matched analysis included 263 pairs of patients. The distribution of propensity scores appeared to be similar in both groups. Furthermore, the average propensity score was also similar [mean (standard deviation) 0.446 (0.161) in registrars and 0.435 (0.155) in nurse practitioners] in each group. A comparison of propensity-matched groups with regard to baseline characteristics and the characteristics of MET calls and the standardized differences are presented in Tables 1 and 2, respectively. Primary and secondary outcomes were compared between the matched pairs, and the results are presented in Table 5. There was no difference in the primary composite outcome between NP- and ICUR-led MET calls. However, the risk of hospital mortality was lower in NP-led MET call group (OR 0.57, 95% CI 0.35–0.91, *p* = 0.02). The likelihood of discharge home was also higher in NP-led MET calls as compared to ICUR-led MET (OR 1.55, 95% CI 1.09–2.2, *p* = 0.015). NP-led MET calls showed a trend toward decreased mortality within 24 h of index MET call, but this did not reach statistical significance (3.4% vs. 7.2%, 0.47 (0.21–1.05); *p* = 0.06).

**Table 5:** Propensity score-matched analysis

	NP led (*n* = 263)	ICU led (*n* = 263)	Odds ratio (95% CI)	*p* value
Composite outcome *n* (%)	56 (21.3)	69 (26.2)	0.77 (0.52–1.14)	0.19
Hospital mortality *n* (%)	33 (12.5)	53 (20.2)	0.57 (0.35–0.91)	0.02
Mortality within 24 h *n* (%)	9 (3.4)	19 (7.2)	0.47 (0.21–1.05)	0.06
Hospital length of stay	109.5	120.7	0.99 (0.98–1.01)	0.48
Discharge home *n* (%)	148 (56.2)	120 (45.6)	1.55 (1.09–2.2)	0.015

Propensity score matched for age, CCI, weekend occurrence, out of hours occurrence, not for resuscitation plan prior to MET call, trigger being drop in oxygen saturation and medical versus surgical diagnosis

#### Multivariable analysis

On multivariable analysis, neither NP- nor ICUR-led MET calls were independently associated with composite outcome. However, ICUR-led MET calls were independently associated with increased hospital mortality (OR 1.74, 95% CI 1.04–2.91; *p* = 0.035).

### Sensitivity analysis

#### Comparison of MET call characteristics and outcomes after excluding weekend and after hours MET calls

Comparison of baseline characteristics is presented in Additional file 1: E-Table 1. There were more number of female patients in ICUR-led MET calls. The rest of the variables compared did not show a statistically significant difference (Additional file 1: E-Table 1). The triggers for MET call were comparable between both groups but for obstructed/threatened airway trigger (Additional file 1: E-Table 2). The interventions recoded during MET calls and the outcomes were comparable between ICUR- and NP-led MET calls (Additional file 1: E-Tables 3, 4).

#### Comparison of MET call characteristics and outcomes of MET calls during weekend and after hours

Comparison of baseline characteristics revealed no significant differences between NP- and ICUR-led MET calls (Additional file 1: E-Table 5). The triggers for MET call were comparable between both groups but for oxygenation saturation trigger (Additional file 1: E-Table 6). The interventions performed differed significantly in terms of IV cannulation, fluid bolus administration and performing ECGs. All these interventions were recorded in a higher proportion in NP-led MET calls (Additional file 1: E-Table 7). The comparison of outcomes showed no difference in composite endpoint, but a higher proportion of ICU admissions within 24 h (38% vs. 30%; *p* < 0.0001), higher mortality within 24 h (8%Vs 3.2%; *p* = 0.005) and higher hospital mortality (21.3% vs. 12.6%; *p* = 0.003) in ICUR-led MET calls (Additional file 1: E-Table 8).

## Discussion

This study aimed to compare clinical outcomes between NP- and ICUR-led MET calls. In the clinical outcomes studied, this study showed no difference in acute deterioration, but a reduction in hospital mortality, and an increased likelihood of discharge home in NP-led MET calls. To our knowledge, NPMET is the first study that directly compared clinically important patient-centered outcomes between NP- and ICUR-led MET calls. Given the retrospective nature of this study, propensity matching was performed to avoid the influence of confounders between the groups. The balance of covariates after propensity matching confirms that both groups are comparable. All other studies either used a retrospective before and after design, focused on recognition of systemic inflammatory response syndrome, measured post-ICU discharge interventions instead of MET call leadership, did not have ICUR-led MET calls for comparison or measured active surveillance for deteriorating patients rather than MET call leadership [9–15].

The organization and governance of MET teams in different organizations are variable and are largely dependent on the availability of personnel [5]. To the best of our knowledge, there are no studies that directly compared the service delivery of NP leading MET calls on clinical outcomes. NPMET study showed that a NP-led model of care in MET calls might offer better clinical outcomes than ICUR-led MET calls. There could be several reasons for the association of NP-led MET calls with lower risk of hospital mortality and higher probability of discharge to home. NPs leading MET calls had prior experience of attending MET calls for over 7 years. Although they were not leading those MET calls, this experience could have contributed to better patient management that may have led to improvement in clinical outcomes. ICUR-led MET calls were attended by registrars with varying experience in assessing and treating acutely ill or deteriorating patients in an unfamiliar ward environment. This is unavoidable in the existing system, where ICURs are expected to gain competence in managing MET calls, while extrapolating knowledge of management of deteriorating patients from the ICU to a ward environment.

It is possible that the differences in the outcomes could also be due to differences in interventions that were performed during the MET calls. Interventions such as IV cannulations, administration of fluid bolus, and performance of ECGs were significantly higher in NP-led MET calls. While the retrospective nature of our study does not allow us to further define the influence of these interventions on the outcomes, it is possible that early correction of the physiological abnormalities during an acute deterioration could have contributed to better outcomes in these MET calls [16]. The other reason may be that most of the NP-led MET calls occurred during business hours where an intensive care consultant was physically present within the hospital. A higher proportion of ICUR-led MET calls occurred out of hours when there was no onsite consultant and this could have negatively affected the outcome.

Furthermore, as NPs are exclusively rostered for liaison between ICU services and ward-based teams, their awareness of the environment and knowledge of available resources during crises can better direct patient management during and after MET calls. A recent in-depth interview study exploring human factors for successful airway management found that knowledge of equipment location and storage, experience and learning, teamwork and communication can all have a positively influence [17]. Additionally, it has been shown that leadership can be spontaneously shared in emergency team scenarios [18]. This sharing of leadership is facilitated by familiarity of ward staff with NPs. ICURs on the other hand are rostered primarily for management of patients in ICU. They rotated between hospitals as part of their training requirement. Hence, their knowledge of the environment, resources and location of equipment may not be comparable with exclusively rostered NPs, despite having knowledge and skills to perform within ICU successfully. NPMET study revealed that MET call leadership by well-trained and qualified NPs familiar with environment, equipment and resourcing can have equivalent and potentially better outcomes than the widely prevalent model of ICURs-led MET calls in a setting similar to that of the study. While availability of well-trained ICU registrars may be variable, it is reassuring to know that a NP-led MET call service can support vulnerable patients deteriorating outside ICU. This knowledge can encourage organizations to plan and support uptake of NP leadership in MET calls, facilitating further prospective analyses.

### Strengths and limitations

#### Strengths

To the best of our knowledge, NPMET is the first study that directly compared clinical outcomes between MET calls led by NP and ICUR. This study had a large sample size of over 1300 MET calls that has enabled us to identify important differences in outcomes between NP- and ICUR-led MET calls. In spite of the retrospective nature of this study, data were largely available on the reasons for MET calls, timing and the interventions that were performed during MET and the outcomes we assessed. The propensity matching of our patients provided good balance of the variables including the timing of MET calls, triggers and comorbidities that could influence the outcome. This facilitated a robust comparison of the differences in the characteristics, interventions performed during the MET calls and the outcomes of MET calls led by NPs and ICUR.

#### Limitations

Despite using propensity matching to ensure MET calls in the analysis are comparable between NP led and ICURs, there may be unknown confounders. Delay in activation of rapid response calls of 15 min or more is known to be associated with higher risk of hospital mortality [19]. We could not record the number of MET calls which involved delayed activation as these data were not recorded on MET call documentation template. Furthermore, we did not have the data on the assistance provided by the consultant in these MET calls and the time spent by the ICUR and NPs on MET calls. As NPMET is a single-center study, selection bias because of our hospital population not being representative of hospital populations of other healthcare services is a possibility. Rapid response systems are heavily dependent on the context of case mix, staffing ratios, clinical support and policy framework. The generalizability of our findings is limited to hospital settings and case mix similar to that of healthcare service studied. We could not perform a sample size calculation prior to data collection, as the number of eligible MET calls was unknown because of retrospective design. We attempted to adjust the confounding effect of the timing of MET calls by using in hours versus out of hours occurrence as a covariate in the regression models. However, residual confounding effect cannot be ruled out.

## Conclusion

NPMET study showed that the primary composite outcome comprising of recurrence of MET call, occurrence of code blue or admission to ICU, all within 24 h, was not different between NP-led and ICUR-led MET calls. NP-led MET calls were associated with lower hospital mortality and higher likelihood of discharge home. Multi-center studies comparing NP and ICUR/physician-led MET calls may further help in generalizing the findings of our study and identify the optimal organization of MET teams for clinical and cost-effectiveness.

## Supplementary Information

**Additional file 1.** Supplementary Information—E Tables 1–8.

## Data Availability

The datasets used and/or analyzed during the current study are available from the corresponding author on reasonable request. All data generated or analyzed during this study are included in this published article.

## Abbreviations

APACHE: Acute Physiology and Chronic Health Evaluation
CCI: Charlson Comorbidity Index
CI: Confidence interval
ICUR: Intensive care unit registrar
IQR: Interquartile range
MET: Medical emergency teams
NP: Nurse practitioners
OR: Odds ratio
SBP: Systolic blood pressure
SD: Standard deviation
