# Supplementary material for: Comparison of clinical outcomes between nurse practitioner and registrar-led medical emergency teams: a propensity-matched analysis
Source: Crit Care. 2021 Mar 22;25:117. doi: 10.1186/s13054-021-03534-4 (PMC7986296; doi:10.1186/s13054-021-03534-4)
Supplement: Supplementary file 1 — Additional file 1. Supplementary Information—E Tables 1–8. [file 13054_2021_3534_MOESM1_ESM.docx]

| **ETable 1. Comparison of baseline characteristics between NP and ICUR led MET calls excluding weekend and afterhours MET calls** | | | | | | |
| --- | --- | --- | --- | --- | --- | --- |
|  | **All** | **NP led (n=635)** | | **ICUR led (n=24)** | | **p Value** |
| Age (SD) | 659 | 635 | 70.1 (18) | 24 | 69.5 (14.4) | 0.87 |
| Female (n) | 659 | 635 | 52.8% (335) | 24 | 70.8% (17) | 0.08 |
| **Admission diagnosis system** |  |  |  |  |  |  |
| Respiratory System | 659 | 635 | 15.9% (101) | 24 | 20.8% (5) | 0.52 |
| Cardiovascular System | 659 | 635 | 13.2% (84) | 24 | 20.8% (5) | 0.29 |
| Nervous System | 659 | 635 | 13.7% (87) | 24 | 20.8% (5) | 0.32 |
| Gastrointestinal System | 659 | 635 | 16.4% (104) | 24 | 8.3% (2) | 0.29 |
| Genitourinary System | 659 | 635 | 8.5% (54) | 24 | 4.2% (1) | 0.45 |
| Other | 659 | 635 | 32.3% (205) | 24 | 25% (6) | 0.45 |
| **Diagnostic category** |  |  |  |  |  |  |
| Medical (%) | 656 | 633 | 61.8% (391) | 23 | 69.6% (16) | 0.45 |
| Surgical (%) | 656 | 633 | 35.5% (225) | 23 | 30.4% (7) | 0.62 |
| Obstetrics and Gynaecology (%) | 656 | 633 | 2.7% (17) | 23 | 0% (0) | 1.00 |
| Charlson Comorbidity Index (IQR) | 659 | 635 | 1 [0-2] | 24 | 1 [0-2] | 0.71 |

| **ETable 2. Comparison of characteristics of MET calls and patients admitted to ICU excluding weekend and afterhours MET calls** | | | | | | | | |  |
| --- | --- | --- | --- | --- | --- | --- | --- | --- | --- |
|  | **All** | **NP led cohort** | | **ICUR led cohort** | | | **p Value** | |  |
|  |  | N | % or mean/median (SD or IQR) | | N | % or mean/median (SD or IQR) | |  | |
| **Trigger** |  |  |  | |  |  | |  | |
| Altered conscious state | 647 | 623 | 14.1% (88) | | 24 | 16.7% (4) | | 0.73 | |
| Arrythmia | 647 | 623 | 0.321% (2) | | 24 | 4.2% (1) | | 0.11 | |
| Heart rate < 40/min | 647 | 623 | 0.642% (4) | | 24 | 0% (0) | | 1.00 | |
| Heart rate > 130/min | 647 | 623 | 14.4% (90) | | 24 | 20.8% (5) | | 0.39 | |
| Obstructed/threatened airway | 647 | 623 | 0.642% (4) | | 24 | 8.3% (2) | | 0.018 | |
| Staff concern | 647 | 623 | 10.4% (65) | | 24 | 12.5% (3) | | 0.75 | |
| Respiratory distress | 647 | 623 | 5.3% (33) | | 24 | 4.2% (1) | | 0.81 | |
| Respiratory rate < 8/min | 647 | 623 | 0.642% (4) | | 24 | 0% (0) | | 1.00 | |
| Respiratory rate > 30 or 36/min | 647 | 623 | 4.8% (30) | | 24 | 0% (0) | | 0.62 | |
| Oxygen saturation < 90% | 647 | 623 | 11.2% (70) | | 24 | 8.3% (2) | | 0.66 | |
| SBP < 90 mm Hg | 647 | 623 | 20.9% (130) | | 24 | 12.5% (3) | | 0.32 | |
| SBP > 180 or 200 mm Hg | 647 | 623 | 13.6% (85) | | 24 | 12.5% (3) | | 0.87 | |
| Seizures | 647 | 623 | 2.2% (14) | | 24 | 0% (0) | | 1.00 | |
| Urine output less than 50 ml/4 hours | 647 | 623 | 0.642% (4) | | 24 | 0% (0) | | 1.00 | |
| **Observations at onset of MET call** | | | | | | | | |  |
| Heart rate (SD) | 540 | 521 | 95.5 (30.3) | | 19 | 106 (35) | | 0.14 | |
| Respiratory rate (SD) | 345 | 332 | 20 [18-27.5] | | 13 | 18 [16-19] | | 0.008 | |
| SBP (SD) | 537 | 518 | 130 (41.3) | | 19 | 150 (42) | | 0.035 | |
| Oxygen saturation (SD) | 491 | 476 | 93.2 (8.59) | | 15 | 93.3 (10.6) | | 0.96 | |
| GCS (IQR) | 22 | 22 | 13 (8-15) | | 0 | . (.) | | 1.00 | |
| Hospital LOS prior to MET call | 656 | 632 | 1.85 [0.707-4.58] | | 24 | 2.51 [0.7-6.93] | | 0.45 | |
| Hospital LOS < 24 hours prior to MET call | 659 | 635 | 65.2% (414) | | 24 | 58.3% (14) | | 0.49 | |
| NFR documented prior to MET call | 657 | 633 | 29.9% (189) | | 24 | 20.8% (5) | | 0.34 | |
| **Patients admitted to ICU within 24 hours of MET call** | | | | | | | | |  |
| APACHE II score | 54 | 51 | 16.7 (5.78) | | 3 | 18.7 (6.66) | | 0.58 | |
| APACHE III score | 54 | 51 | 60.6 (21.9) | | 3 | 57.3 (10) | | 0.8 | |
| ICU LOS | 55 | 52 | 2.51 [1.13-4.61] | | 3 | 1.93 [1.14-2.39] | | 0.54 | |
| ICU mortality | 55 | 52 | 9.6% (5) | | 3 | 0% (0) | | 1.00 | |

| **ETable 3. Interventions recorded during MET calls** | | | | | | |
| --- | --- | --- | --- | --- | --- | --- |
|  | **All** | **NP led (n = 635)** | | **ICUR led (n=24)** | | **p Value** |
| IV cannulation | 659 | 635 | 25.4% (161) | 24 | 37.5% (9) | 0.18 |
| Fluid Bolus | 659 | 635 | 32.6% (207) | 24 | 25% (6) | 0.44 |
| ECG | 659 | 635 | 61.4% (390) | 24 | 45.8% (11) | 0.13 |
| Arterial blood gas analysis | 659 | 635 | 23.3% (148) | 24 | 16.7% (4) | 0.45 |
| Body fluid or blood cultures | 659 | 635 | 11.3% (72) | 24 | 8.3% (2) | 0.65 |
| Bag and Mask ventilation | 659 | 635 | 0.157% (1) | 24 | 0% (0) | 1.00 |
| Endotracheal intubation | 659 | 635 | 0% (0) | 24 | 0% (0) | 1.00 |
| Change in resuscitation plans after MET call | 654 | 630 | 17.3% (109) | 24 | 8.3% (2) | 0.25 |

| **ETable 4. Comparison of outcomes between NP led vs ICU led MET calls after excluding afterhours and weekend MET calls** | | | | | | |
| --- | --- | --- | --- | --- | --- | --- |
|  | **All** | **NP led (n=635)** | | **ICUR led (n=24)** | | **p Value** |
| **Primary Outcome** |  |  |  |  |  |  |
| **Composite endpoint** | 659 | 635 | 19.4% (123) | 24 | 20.8% (5) | 0.86 |
| *Components of composite outcome* | | | | | | |
| At least one MET call within 24hrs | 659 | 635 | 13.5% (86) | 24 | 8.3% (2) | 0.46 |
| Code Blue within 24 hours | 659 | 635 | 0% (0) | 24 | 0% (0) | 1.00 |
| ICU admission within 24 hours | 659 | 635 | 8.2% (52) | 24 | 12.5% (3) | 0.45 |
| **Secondary Outcomes** | | | | | | |
| Mortality within 24 hours | 659 | 635 | 3.5% (22) | 24 | 4.2% (1) | 0.85 |
| Hospital mortality | 659 | 635 | 12.8% (81) | 24 | 12.5% (3) | 0.97 |
| Hospital Length of stay | 659 | 635 | 7.5 [4.04-14] | 24 | 8.46 [4.68-16.8] | 0.57 |
| Discharged home | 659 | 635 | 48.5% (308) | 24 | 50% (12) | 0.89 |
|  |  |  |  |  |  |  |
|  |  |  |  |  |  |  |
|  |  |  |  |  |  |  |

| **ETable 5. Comparison of baseline characteristics between NP and ICUR led MET calls – afterhours and weekend MET calls only** | | | | | | |
| --- | --- | --- | --- | --- | --- | --- |
|  | **All** | **NP led (n=435)** | | **ICUR led (n=249)** | | **p Value** |
| Age | 684 | 435 | 68.2 (18.7) | 249 | 67.5 (19.6) | 0.65 |
| Female | 684 | 435 | 52.6% (229) | 249 | 54.6% (136) | 0.62 |
| **Admission diagnosis system** | | | | | | |
| Nervous_System | 684 | 435 | 15.4% (67) | 249 | 14.9% (37) | 0.85 |
| Gastrointestinal_System | 684 | 435 | 16.6% (72) | 249 | 14.5% (36) | 0.47 |
| Genirourinary_System | 684 | 435 | 8.5% (37) | 249 | 7.6% (19) | 0.69 |
| Other | 684 | 435 | 32% (139) | 249 | 29.7% (74) | 0.54 |
| **Diagnostic Category** |  |  |  |  |  |  |
| Medical | 683 | 435 | 65.7% (286) | 248 | 71.4% (177) | 0.13 |
| Surgical | 683 | 435 | 31.7% (138) | 248 | 27.4% (68) | 0.24 |
| Obstetrics and Gynaecology | 683 | 435 | 2.5% (11) | 248 | 1.2% (3) | 0.24 |
| Charlson Comorbidity Index (IQR) | 684 | 435 | 1 [0-3] | 249 | 1 [0-3] | 0.25 |
|  |  |  |  |  |  |  |
|  |  |  |  |  |  |  |
|  |  |  |  |  |  |  |

| **ETable 6. Comparison of characteristics of MET calls and patients admitted to ICU - weekend and afterhours MET calls only** | | | | | | | | |  |
| --- | --- | --- | --- | --- | --- | --- | --- | --- | --- |
|  | **All** | **NP led cohort** | | **ICUR led cohort** | | | **p Value** | |  |
|  |  | N | % or mean/median (SD or IQR) | | N | % or mean/median (SD or IQR) | |  | |
| **Trigger** |  |  |  | |  |  | |  | |
| Altered conscious state | 675 | 433 | 12.9% (56) | | 242 | 8.7% (21) | | 0.1 | |
| Arrythmia | 675 | 433 | 0.231% (1) | | 242 | 0.413% (1) | | 1.00 | |
| Heart rate < 40/min | 675 | 433 | 0.462% (2) | | 242 | 0.826% (2) | | 0.62 | |
| Heart rate > 130/min | 675 | 433 | 19.9% (86) | | 242 | 16.9% (41) | | 0.35 | |
| Obstructed/threatened airway | 675 | 433 | 0.231% (1) | | 242 | 0.413% (1) | | 1.00 | |
| Staff concern | 675 | 433 | 11.5% (50) | | 242 | 9.5% (23) | | 0.41 | |
| Respiratory distress | 675 | 433 | 7.4% (32) | | 242 | 9.1% (22) | | 0.44 | |
| Respiratory rate < 8/min | 675 | 433 | 0% (0) | | 242 | 0% (0) | | 1.00 | |
| Respiratory rate > 30 or 36/min | 675 | 433 | 3.5% (15) | | 242 | 2.9% (7) | | 0.69 | |
| Oxygen saturation < 90% | 675 | 433 | 8.1% (35) | | 242 | 15.7% (38) | | 0.002 | |
| SBP < 90 mm Hg | 675 | 433 | 19.2% (83) | | 242 | 21.1% (51) | | 0.55 | |
| SBP > 180 or 200 mm Hg | 675 | 433 | 13.4% (58) | | 242 | 9.5% (23) | | 0.14 | |
| Seizures | 675 | 433 | 3% (13) | | 242 | 4.1% (10) | | 0.44 | |
| Urine output less than 50 ml/4 hours | 675 | 433 | 0.231% (1) | | 242 | 0.826% (2) | | 0.29 | |
| **Observations at onset of MET call** | | | | | | | | |  |
| Heart rate (SD) | 527 | 348 | 104 (30.7) | | 179 | 105 (36.2) | | 0.78 | |
| Respiratory rate (SD) | 393 | 262 | 20 [16-28] | | 131 | 22 [18-30] | | 0.03 | |
| SBP (SD) | 522 | 351 | 132 [99-171] | | 171 | 120 [90-153] | | 0.028 | |
| Oxygen saturation (SD) | 489 | 319 | 92.6 (7.22) | | 170 | 90.6 (9.38) | | 0.009 | |
| GCS (IQR) | 24 | 17 | 12.1 (4.45) | | 7 | 12.4 (3.74) | | 0.85 | |
| Hospital LOS prior to MET call | 680 | 431 | 1.63 [0.553-3.87] | | 249 | 1.73 [0.597-4] | | 0.48 | |
| Hospital LOS < 24 hours prior to MET call | 684 | 435 | 61.8% (269) | | 249 | 65.5% (163) | | 0.35 | |
| NFR documented prior to MET call | 683 | 435 | 30.3% (132) | | 248 | 34.7% (86) | | 0.24 | |
| **Patients admitted to ICU within 24 hours of MET call** | | | | | | | | |  |
| APACHE II score | 66 | 30 | 17.7 (6.79) | | 36 | 15.9 (6.53) | | 0.26 | |
| APACHE III score | 66 | 30 | 63.1 (25.1) | | 36 | 61.6 (21.8) | | 0.79 | |
| ICU LOS | 68 | 30 | 1.83 [1.11-3.15] | | 38 | 2.64 [1.34-3.54] | | 0.43 | |
| ICU mortality | 68 | 30 | 6.7% (2) | | 38 | 7.9% (3) | | 1.00 | |

| **ETable 7. Interventions recorded during MET calls – Afterhours and weekend MET calls only** | | | | | | |
| --- | --- | --- | --- | --- | --- | --- |
|  | **All** | **NP led (n = 435)** | | **ICUR led (n=249)** | | **p Value** |
| IV cannulation | 682 | 434 | 22.4% (97) | 248 | 13.3% (33) | 0.004 |
| Fluid Bolus | 682 | 434 | 34.6% (150) | 248 | 26.6% (66) | 0.032 |
| ECG | 681 | 435 | 54.5% (237) | 246 | 42.3% (104) | 0.002 |
| Arterial blood gas analysis | 680 | 435 | 17% (74) | 245 | 22% (54) | 0.11 |
| Body fluid or blood cultures | 683 | 435 | 13.6% (59) | 248 | 8.1% (20) | 0.031 |
| Bag and Mask ventilation | 684 | 435 | 0% (0) | 249 | 0.402% (1) | 0.36 |
| Endotracheal intubation | 684 | 435 | 0% (0) | 249 | 0.402% (1) | 0.36 |
| Change in resuscitation plans after MET call | 675 | 430 | 15.1% (65) | 245 | 14.3% (35) | 0.77 |

| **ETable 8. Comparison of outcomes between NP led vs ICU led MET calls - Afterhours and weekend MET calls** | | | | | | |
| --- | --- | --- | --- | --- | --- | --- |
|  | **All** | **NP led (n=435)** | | **ICUR led (n=249)** | | **p Value** |
| **Primary Outcome** |  |  |  |  |  |  |
| **Composite endpoint** | 684 | 435 | 22.3% (97) | 249 | 27.3% (68) | 0.14 |
| *Components of composite outcome* | | | | | | |
| At least one MET call within 24hrs | 684 | 435 | 17.7% (77) | 249 | 15.7% (39) | 0.49 |
| Code Blue within 24 hours | 684 | 435 | 0.69% (3) | 249 | 0.402% (1) | 1.00 |
| ICU admission within 24 hours | 684 | 435 | 6.9% (30) | 249 | 15.3% (38) | <0.0001 |
| **Secondary Outcomes** | | | | | | |
| Mortality within 24 hours | 684 | 435 | 3.2% (14) | 249 | 8% (20) | 0.005 |
| Hospital mortality | 684 | 435 | 12.6% (55) | 249 | 21.3% (53) | 0.003 |
| Hospital Length of stay | 684 | 435 | 7.61 [4.18-14.8] | 249 | 7.08 [3.7-13.9] | 0.76 |
| Discharged home | 684 | 435 | 52.9% (230) | 249 | 45% (112) | 0.047 |
